# Risk factors of uterine contraction after ureteroscopy in pregnant women with renal colic

**DOI:** 10.1007/s11255-021-02932-5

**Published:** 2021-07-05

**Authors:** Chunjing Li, Liwen Guo, Mi Luo, Mingjuan Guo, Jierong Li, Shilin Zhang, Guoqing Liu

**Affiliations:** 1 Department of Urology, Affiliated Foshan Maternal and Child Healthcare Hospital, Southern Medical University, Foshan, Guangdong 528000 People’s Republic of China; 2 Department of Gynecology, Ningde Municipal Hospital, Fujian Medical University, Ningde, Fujian 352100 People’s Republic of China

**Keywords:** Pregnant women, Renal colic, Ureteroscopy, Uterine contraction, Risk factors

## Abstract

**Background:**

Ureteroscopy is widely applied in pregnant women with renal colic, but such patients are easy to experience uterine contraction after surgery. There are many factors which may affect uterine contraction, this study aims to explore the risk factors of uterine contraction triggered by ureteroscopy in pregnant women with renal colic.

**Methods:**

One hundred and one pregnant women were retrospectively analyzed, the patients were hospitalized because of severe renal colic. All patients received ureteroscopy during which double J catheters were inserted into ureters for drainage. Patients received other medical treatments individually according to their condition and uterine contractions were detected by EHG within 12 h after operation. Patients were classified as group A (uterine contraction) and group B (no uterine contraction) according to the presence or absence of continuously regular uterine contraction. Clinical characteristics were collected for further analysis, including history of childbirth, anesthesia method, application of phloroglucinol or not, operation time, Oxygen inhalation or not, pain relief or not after surgery, systemic inflammatory response syndrome (SIRS) occurred or not. A binary logistic regression analysis model was established to explore whether such clinical characteristics were relevant to uterine contraction after ureteroscopy.

**Results:**

Continuously regular uterine contraction presented in 46 pregnant women within 12 h after ureteroscopy, making the incidence of uterine contraction as high as 45.54%. The presence of uterine contraction was related to the following factors(*P* < 0.05): history of childbirth (primipara versus multipara)(OR 6.593, 95% CI 2.231–19.490), operation time (each quarter additional) (OR 2.385, 95% CI 1.342–4.238), application of phloroglucinol (yes versus not) (OR 6.959, 95% CI 1.416–34.194), pain relief after surgery(yes versus not)(OR 6.707, 95% CI 1.978–22.738), SIRS occurred after surgery (yes versus not) (OR 0.099, 95% CI 0.014–0.713).

**Conclusion:**

Continuously regular uterine contraction is easy to occur within 12 h after ureteroscopy in pregnant women. SIRS occurred after surgery is a risk factor for uterine contraction; on the contrary, no history of childbirth, shorter operation time, application of phloroglucinol, pain relief after surgery are protective factors.

## Background

Renal colic is one of the most common non obstetric pain and pregnancy emergencies in pregnant women [1]. The incidence range from 1:200 to 1:2000. For pregnant women, renal colic could lead to severe pregnancy complications, such as abortion or premature delivery [2]. The main cause of renal colic in pregnant women contains urinary calculi, ureteral stricture, and ureteral obstruction caused by external compression and so on. Spasmolysis, pain relief, double-J tube drainage or percutaneous nephrostomy are options considered to be effective for renal colic [3]. Due to the limitation of drug application for pregnant women and the trauma caused by nephrostomy [4, 5], thereby ureteroscopy is widely considered to be a preferable choice. Uncontrollable pain and gradually aggravating ureteral obstruction are surgical indications for ureteroscopy [6, 7]. Complications such as urinary tract infection and so on are common after ureteroscopy, but premature delivery or abortion derived from regular uterine contraction is one of the most severe complications [8, 9]. Persistent and intense uterine contraction, which could lead to cervical dilatation, is a sign of threatened preterm birth [10], even if the length of cervical canal changes little [11], the premature birth rate of pregnant women with renal colic could be as high as 12.8% according to previous literatures [12]. Therefore, preventing the occurrence of continuously regular uterine contraction is the premise to avoid adverse pregnancy events. The occurrence of uterine contraction is interactive result of multiple factors [13]. However, there is little literature about risk factors of uterine contraction caused by ureteroscopy. In this study, we retrospectively analyzed 101 pregnant women who were received ureteroscopy to explore the risk factors of uterine contraction after such surgery.

## Methods

One hundred and one pregnant women who received ureteroscopy in our hospital from January 2010 to Mar 2020 were retrospectively analyzed, including 46 primiparas and 55 multipara. The patients, at 20–36 weeks of gestation, were admitted because of severe renal colic. The mean duration of hospital stay was 5.92 ± 1.83 day. The diseases included ureteral calculi (62 cases), renal calculus (10 cases), ureteral stricture (18 cases) and ureteral obstruction (11 cases) caused by fetal oppression. The cervical was not opened and there was no continuously regular uterine contraction detected by EHG before ureteroscopy. A visual analog scale/score (VAS) was conduct to evaluate the pain degree, if the score was more than 7, then phloroglucinol 80 mg/single-use or pethidine 75–100 mg/single-use was prescribed for every patient. If the renal colic still had little relief or occurred again in less than 8 h, then patients would receive ureteroscopy (rigid ureteroscope, Wolf, 9.8Fr, Richard-wolf, Germany) and double-J catheters(6Fr, Boston scientific, MA, US) drainage on the basis of informed consent. In fact, for all the 101 patients before surgery and 26 patients among them after surgery, the VAS scores were all more than 7. The anesthesia methods included general anesthesia (13 cases), lumbar anesthesia (39 cases) and spinal-epidural anesthesia (49 cases). After operation, EHG was used again to monitor continuously regular uterine contraction for 12 h. Phloroglucinol was applied in 42 patients; Oxygen inhalation was given to 61 patients; Renal colic was not completely relieved in 26 patients immediately after surgery but disappeared after several hours; Systemic inflammatory response syndrome (SIRS) occurred in 11 patients but nobody progressed to Sepsis. According to the occurrence of continuously regular uterine contraction after operation, the patients were divided into group A and group B. Group A (*n* = 46) presented regular contractions within 12 h after surgery and received magnesium sulfate or ritodrine hydrochloride, while group B (*n* = 55) did not present regular contractions within 12 h after ureteroscopy. Patients’ differences in age, weight and gestational week between the two groups were not statistically significant (*P* > 0.05) (Tab 1).

**O:** Clinical characteristics of patients in two groups

Group	Age (Y)	Weight (Kg)	Gestational week (W)	Operation time (min)	Disease species (case)			
					Ureterolith	RS	US	UO
A (*n* = 46)	28.0 ± 5.7	58.0 ± 8.3	26.4 ± 4.4	28.8 ± 16.3*	23	5	13	5
B (*n* = 55)	29.8 ± 5.2	61.6 ± 9.6	25.8 ± 4.7	23.8 ± 11.1	39	5	5	6

*RS* renal stone, *US* ureteral stricture, *UO* ureteral obstruction

^*^P < 0.05

Diagnosis and treatment criteria were as follows: the uterine contraction were monitored continuously after admission until 12 h after operation. Any uterine contraction lasted for more than 30 s with a interval less than 15 min would be considered as real uterine contraction [14]. If the length of cervical canal shortened, 25% magnesium sulfate or 0.01% ritodrine hydrochloride was prescribed immediately to inhibit uterine contraction. In view of the side effects of uterine contraction inhibitors, the following indicators were needed to monitor closely during the application of such drugs. (1) Breath frequency no less than 16 times/min; (2) heart rate no more than 120 times/min; (3) knee reflex existed; (4) urine volume not less than 17 ml/h [15]. The diagnostic criteria of systemic inflammatory response syndrome were as follows: (1) body temperature > 38.0 ℃ or body temperature < 36.0 ℃; (2) heart rate > 90 beats/min; (3) respiration rate > 20 beats/min or excessive breathing with PaCO_2_ < 32 mmHg; (4) blood WBC > 12.0 × 10^9^/L, or WBC < 4.0 × 10^9^/L or immature WBC > 10.0% [16].

Statistics: in addition to age, weight and gestational weeks, the other clinical characteristics of patients in the two groups were collected, including history of childbirth, anesthesia method, operation time (each quarter additional), phloroglucinol application or not, oxygen inhalation or not, renal colic relief or not, and SIRS occurrence or not. The above clinical features were analyzed to explore whether they were related to the presence of uterine contraction (Table 2).

**Table 2:** Clinical characteristics for logistics analysis

Clinical characteristics	Group A (case)	Group B (case)
History of childbirth		
Primipara	30	16
Multipara	16	39
Anesthesia methods		
General anesthesia	7	6
Lumbar anesthesia	19	20
Spinal-epidural anesthesia	20	29
Operation time (min)		
≤ 15	9	17
> 15 ≤ 30	22	25
> 30 ≤ 45	6	11
> 45 ≤ 60	6	2
> 60 ≤ 75	3	0
Application of phloroglucinol		
Yes	36	49
No	10	6
Oxygen inhalation		
Yes	31	28
No	15	27
Complete relief of renal colic		
Yes	26	49
No	20	6
Occurrence of SIRS		
Yes	9	2
No	37	53

All statistical analyses were performed by SPSS version 23.0 (IBM, New York, NY). Measurement data were showed as mean ± standard deviation (x ± s), rate or percentage were compared by Chi-squared test. A binary logistic regression model was established to explore the clinical characteristics which might be related to uterine contraction, adjusted odds ratio (OR) and 95% confidence interval (CI) were analyzed. The *P* value less than 0.05 was considered to be statistically significant.

## Result

In all 101 patients, continuously regular uterine contractions which could be inhibited by magnesium sulfate or ritodrine occurred in 46 patients within 12 h after operation. No abortion or premature delivery occurred in any patient. Binary logistic regression analysis showed that history of childbirth, operation time, phloroglucinol application, relief of renal colic and SIRS occurrence were related to the presence of uterine contraction (*P* < 0.05). According to the statistical results, presence of SIRS after surgery (OR 0.099, 95% CI 0.014–0.713) was the risk factor for uterine contraction, meanwhile, no history of childbirth (OR 6.593, 95% CI 2.231–19.490), shorter operation time(each quarter additional) (OR 2.385, 95% CI 1.342–4.238), phloroglucinol application (OR 6.959, 95% CI 1.416–34.194), complete relief of renal colic(OR 6.707, 95% CI 1.978–22.738) were protective factors for uterine contraction (Table 3).

**Table 3:** Risk factors for uterine contraction in pregnant women after ureteroscopy

Risk factors	OR	95% CI	*P*
History of deliveries			
Primipara versus multipara	6.593	2.231–19.490	0.001
Duration of surgery			
Short versus long	2.385	1.342–4.238	0.003
Application of phloroglucinol			
Yes versus no	6.959	1.416–34.194	0.017
Pain relief			
Complete versus incomplete	6.707	1.978–22.738	0.002
SIRS			
Occurred versus not occurred	0.099	0.014–0.713	0.022

## Discussion

At present, the definition of preterm birth is still controversial. Flenady et al. [17]. Pointed out that any childbirth within gestational age between 20 and 36 weeks could be defined as Preterm birth which was the main contributor of perinatal morbidity and the second leading cause of neonatal death in the world. The most common clinical symptom of urolithiasis during pregnancy was renal colic and mainly was observed after 20 weeks of gestation [18]. In consideration of ritodrine hydrochloride’s instructions (only for pregnant women after 20 gestational week) and the difficulty for monitoring uterine contractions at early gestation [19], thereby we only retrospective analyzed pregnant women at 20 to 36 gestational week. Johnson et al. [12] had shown that the obstetric complications after ureteroscopy were about 4.0%, but in our study there was nothing severe complication except for that 45.54% of patients developed to continuously regular contractions after ureteroscopy. The incidence of uterine contraction in our study was higher than that reported by other scholars [20]. The possible explanation were as follows: (1) all cases summarized in the study were patients with severe renal colic which could not be relieved by painkiller in outpatient department, in view of the relevance between pain and uterine contraction, the incidence of such complication rose accordingly. (2) all patients were received uterine contraction monitoring by EHG for 12 h after surgery, thus any slight contractions which might be ignored by doctors could be detected in time and analyzed in our study, but in fact, some uterine contractions in early stage could disappear without any medical treatment. There was not any severe complications such as abortion or premature delivery occurred in this study, the results suggested that ureteroscopy was a relatively safe and effective surgical method. The results also reminded us that routine monitoring of uterine contractions after ureteroscopy might help to avoid pregnancy complications.

Compared with primipara, the cervix of multipara is easier to expand, which means a sooner labor process [21, 22]. Literature has shown that uterine contraction was stronger for multipara during labor and more analgesics were needed for painless delivery [23]. Our study showed that multipara were more likely to develop to uterine contraction after ureteroscopy. The possible explanations for this results were as follows: (1) compared with primipara, the oxytocin receptor which played a key role in uterine contraction had a higher expression on uterus in multipara [24, 25]; (2) with the increasing of age, the incidence of pregnancy complications rose in step, multipara was more likely to develop to uterine contraction after operation [26, 27].

Enlarged uterus due to pregnancy inevitably result in ureter compression because of the adjacent location between lower segment ureter and cervix [28–30]. The operation of ureteroscope would inevitably stimulate the cervix, long time and repeatedly physical stimulation could trigger uterine contraction. Scholars had shown that pregnancy complications were prone to occur after long-time surgery [31], and shortening operation time could reduce such complications [32]. Our results also indicated that long operation time was a risk factor for uterine contraction. This study showed that each additional a quarter increased, the adjusted odds ratio was as high as 2.385. This result also reminded us that surgeons should shorten operation time as much as possible when ureteroscopy were performed on pregnant women. The purpose of ureteroscopy was just to resolve urethral obstruction, not for stone free.

Phloroglucinol, as a non-papaverine antispasmodic, only acts on spastic smooth muscles and has minimal impact on normal smooth muscles. Application of phloroglucinol during pregnancy does not increase the risk of fetal malformations [33]. It was applied to relieve renal colic and inhibit uterine contractions for decades [34, 35]. Our research showed that application of phloroglucinol after ureteroscopy could reduce the incidence of uterine contractions. In this study, only a few doctors prescribed phloroglucinol to patients after ureteroscopy, but the results indicated again that such drug may inhibit uterine contraction caused by renal colic for pregnant women.

If severe pain persists, human body would release prostaglandins which could promote cervical ripening and lead to smooth muscle contraction in uterine [36, 37]. Pain could also cause stress response and then trigger an increasing release of catecholamines that plays an important role in uterine contractions [38]. Furthermore, severe renal colic often accompanies with repeated vomit which may lead to uterus compression. Mechanical stimulation could also induces uterus contractions [39]. Our research also showed that unrelieved pain after surgery was a risk factor for uterine contractions. Twenty six patients in this study had little relief in renal colic immediately after ureteroscopy. The result was coincide with other reports [3]. The possible explanations was that the double J catheter inserted into ureter during operation did not play a effective role in drainage soon after surgery. With painkiller prescribed, pain in these patients gradually subsided in 12 h after operation.

During the operation of ureteroscopy, continuously intraureteral perfusion by saline is required. Increasing water pressure in ureter would force bacteria flow back into blood via renal pelvic-vein system, it is more likely to happen in patients with urinary tract infection. Bacteremia may result in spread of inflammation or even progress to SIRS which could develop to multiple organ dysfunction syndrome (MODS) [40]. Inflammatory mediators would be release and spread if SIRS occurs, such mediators could lead to uterus contraction [41, 42]. Our research also suggested that SIRS was a risk factor for uterus contraction. Pregnant women were more likely to develop to sepsis due to immunosuppression and down regulation of inflammatory response because of immunotolerance in pregnancy [43]. pregnant women with SIRS or sepsis may easily be ignored because of unconspicuous fever and quick heart rate caused by ritodrine hydrochloride [44], therefore, it is necessary to closely monitor the infection indicators, such as white blood cells, CRP, and PCT-Q and so on, in order to find potential infection in time and prevent uterus contraction caused by inflammation.

### Limitations

There are also some limitations in this study. First, this study is a single center retrospective study, and the number of patients collected is not adequate enough; Second, there are many factors that may play a role in uterine contraction, such as scar uterus, but it is difficult to collect enough clinical feature by retrospective analysis; Thirdly, in this study, all patients only received uterine contraction monitoring for 12 h by EHG after surgery, but some contractions would not occur within 12 h and would be ignored.

## Conclusion

Ureteroscopy is considered to be a safe and effective treatment for pregnant women, but the high incidence of uterine contraction after surgery still calls for more attention. In order to reduce the odds of uterine contraction after operation, surgeon are supposed to finish the operation as soon as possible, prescribe phloroglucinol after operation, relieve pain and control infection effectively, and pay more attention to multipara.

## Data Availability

The data used and analyzed in this study are available from the corresponding author on reasonable request. The data that support the findings of this study has been upload as attachment in submission.
